# Supplementary material for: Band of mothers: Childbirth as a female bonding experience
Source: PLoS One. 2020 Oct 21;15(10):e0240175. doi: 10.1371/journal.pone.0240175 (PMC7577500; doi:10.1371/journal.pone.0240175)
Supplement: S1 Appendix — (DOCX) [file pone.0240175.s001.docx]

**S1 Appendix. Study Advertisement Posted on Websites.**

Hello!  I am a researcher at the University of Oxford and am doing a study looking at the experience of childbirth for first-time mothers.

This project is funded by a UK grant from the Economic and Social Research Council.  We are interested in seeing whether certain kinds of childbirth experiences for first-time mothers lead to any kinds of feelings with those they believe have shared the experience.  We also want to look at mental health during and after pregnancy.

**Who can participate?**

All first-time mothers should be living in the United States and be above the age of 18.

We are inviting 2 different groups of first-time mothers to participate:

**(1)** Women who are currently pregnant with their firstborn child, and

**(2)** Women who have a firstborn child that is 6 months old or younger.

If you would like to participate, please click on **one** of the two links pasted below, and you will be redirected to an anonymous online questionnaire.

***If you are currently pregnant with your firstborn child, please click here:**

***If you have a firstborn child that is 6 months old or younger, please click here:**

Everyone will be emailed a **$10 Amazon e-gift card** as a thank you for their participation.

Please feel free to contact me if you have any questions:

Thank you so much!  Wishing you a wonderful day!
