# Supplementary material for: Band of mothers: Childbirth as a female bonding experience
Source: PLoS One. 2020 Oct 21;15(10):e0240175. doi: 10.1371/journal.pone.0240175 (PMC7577500; doi:10.1371/journal.pone.0240175)
Supplement: S2 Appendix — (DOCX) [file pone.0240175.s002.docx]

**S2 Appendix. Screening Question for the Antenatal and Postpartum Questionnaires.**

What describes your life stage best? Please choose one of the following options.

Not pregnant and never have given birth to a child

Not currently pregnant, but have given birth to one child

Not currently pregnant, but have given birth to multiple children

Currently pregnant with first child

Currently pregnant with second or later child
