# Supplementary material for: Band of mothers: Childbirth as a female bonding experience
Source: PLoS One. 2020 Oct 21;15(10):e0240175. doi: 10.1371/journal.pone.0240175 (PMC7577500; doi:10.1371/journal.pone.0240175)
Supplement: S3 Appendix — (DOCX) [file pone.0240175.s003.docx]

**S3 Appendix. Adapted Version of the Edinburgh Postnatal Depression Scale for the Antenatal Questionnaire.**

As you are in the first, second, or third trimester of your pregnancy, we would like to know how you are feeling. Please choose the answer which comes closest to how you have felt in the past 7 days, not just how you feel today.

First, here is an example: if you have felt happy most of the time in the past 7 days, then you would choose **Yes, most of the time**, as is also highlighted in light blue below.

I have felt happy:

Yes, all the time

**Yes, most of the time**

No, not very often

No, not at all

This would mean: “I have felt happy most of the time” during the past week. Please complete the other questions in the same way.

In the past 7 days:

1. I have been able to laugh and see the funny side of things

As much as I always could

Not quite so much now

Definitely not so much now

Not at all

2. I have looked forward with enjoyment to things

As much as I ever did

Rather less than I used to

Definitely less than I used to

Hardly at all

3. I have blamed myself unnecessarily when things went wrong

Yes, most of the time

Yes, some of the time

Not very often

No, never

4. I have been anxious or worried for no good reason

No, not at all

Hardly ever

Yes, sometimes

Yes, very often

5. I have felt scared or panicky for no very good reason

Yes, quite a lot

Yes, sometimes

No, not much

No, not at all

6. Things have been getting on top of me

Yes, most of the time I haven’t been able to cope at all

Yes, sometimes I haven’t been coping as well as usual

No, most of the time I have coped quite well

No, I have been coping as well as ever

7. I have been so unhappy that I have had difficulty sleeping

Yes, most of the time

Yes, sometimes

Not very often

No, not at all

8. I have felt sad or miserable

Yes, most of the time

Yes, quite often

Not very often

No, not at all

9. I have been so unhappy that I have been crying

Yes, most of the time

Yes, quite often

Only occasionally

No, never

10. The thought of harming myself has occurred to me

Yes, quite often

Sometimes

Hardly ever

Never
