# Supplementary material for: Band of mothers: Childbirth as a female bonding experience
Source: PLoS One. 2020 Oct 21;15(10):e0240175. doi: 10.1371/journal.pone.0240175 (PMC7577500; doi:10.1371/journal.pone.0240175)
Supplement: S4 Appendix — (DOCX) [file pone.0240175.s004.docx]

**S4 Appendix. Second Screening Question for the Postpartum Questionnaire.**

What age group does your firstborn child fall under? Please choose one of the following options.

0-6 months

7 months-1 year

2-5 years

6 years and above
