# Supplementary material for: Band of mothers: Childbirth as a female bonding experience
Source: PLoS One. 2020 Oct 21;15(10):e0240175. doi: 10.1371/journal.pone.0240175 (PMC7577500; doi:10.1371/journal.pone.0240175)
Supplement: S5 Appendix — (DOCX) [file pone.0240175.s005.docx]

**S5 Appendix. Adapted Version of the Posttraumatic Diagnostic Scale – Self-Report Version for DSM-5 for the Postpartum Questionnaire.**

Below is a list of problems that people sometimes have after experiencing a traumatic event.

Please read each statement carefully and choose the number that best describes how often that problem has been happening and how much it upset you over THE LAST MONTH. Rate each problem with respect to *your childbirth experience.*

First, here is an example: if you’ve talked to a friend about *your childbirth experience* one time in the past month, you would choose **1 – Once a week or less/a little**, as is also highlighted in light blue below (because one time in the past month is less than once a week)

**Talking to other people about *your childbirth experience***

0 – Not at all

**1 – Once a week or less/a little**

2 – 2 to 3 times a week/somewhat

3 – 4 to 5 times a week/very much

4 – 6 or more times a week/severe

1. Unwanted upsetting memories about your childbirth experience

0 – Not at all

1 – Once a week or less/a little

2 – 2 to 3 times a week/somewhat

3 – 4 to 5 times a week/very much

4 – 6 or more times a week/severe

2. Bad dreams or nightmares related to your childbirth experience

0 – Not at all

1 – Once a week or less/a little

2 – 2 to 3 times a week/somewhat

3 – 4 to 5 times a week/very much

4 – 6 or more times a week/severe

3. Reliving your childbirth experience or feeling as if it were actually happening again

0 – Not at all

1 – Once a week or less/a little

2 – 2 to 3 times a week/somewhat

3 – 4 to 5 times a week/very much

4 – 6 or more times a week/severe

4. Feeling very EMOTIONALLY upset when reminded of your childbirth experience

0 – Not at all

1 – Once a week or less/a little

2 – 2 to 3 times a week/somewhat

3 – 4 to 5 times a week/very much

4 – 6 or more times a week/severe

5. Having PHYSICAL reactions when reminded of your childbirth experience (for example, sweating, heart racing)

0 – Not at all

1 – Once a week or less/a little

2 – 2 to 3 times a week/somewhat

3 – 4 to 5 times a week/very much

4 – 6 or more times a week/severe

6. Trying to avoid thoughts or feelings related to your childbirth experience

0 – Not at all

1 – Once a week or less/a little

2 – 2 to 3 times a week/somewhat

3 – 4 to 5 times a week/very much

4 – 6 or more times a week/severe

7. Trying to avoid activities, situations, or places that remind you of your childbirth experience or that feel more dangerous since your childbirth experience

0 – Not at all

1 – Once a week or less/a little

2 – 2 to 3 times a week/somewhat

3 – 4 to 5 times a week/very much

4 – 6 or more times a week/severe

8. Not being able to remember important parts of your childbirth experience

0 – Not at all

1 – Once a week or less/a little

2 – 2 to 3 times a week/somewhat

3 – 4 to 5 times a week/very much

4 – 6 or more times a week/severe

9. Seeing yourself, others, or the world in a more negative way (for example “I can’t trust people,” “I’m a weak person”)

0 – Not at all

1 – Once a week or less/a little

2 – 2 to 3 times a week/somewhat

3 – 4 to 5 times a week/very much

4 – 6 or more times a week/severe

10. Blaming yourself or others (besides those involved in your childbirth experience) for what happened

0 – Not at all

1 – Once a week or less/a little

2 – 2 to 3 times a week/somewhat

3 – 4 to 5 times a week/very much

4 – 6 or more times a week/severe

11. Having intense negative feelings like fear, horror, anger, guilt or shame

0 – Not at all

1 – Once a week or less/a little

2 – 2 to 3 times a week/somewhat

3 – 4 to 5 times a week/very much

4 – 6 or more times a week/severe

12. Losing interest or not participating in activities you used to do

0 – Not at all

1 – Once a week or less/a little

2 – 2 to 3 times a week/somewhat

3 – 4 to 5 times a week/very much

4 – 6 or more times a week/severe

13. Feeling distant or cut off from others

0 – Not at all

1 – Once a week or less/a little

2 – 2 to 3 times a week/somewhat

3 – 4 to 5 times a week/very much

4 – 6 or more times a week/severe

14. Having difficulty experiencing positive feelings

0 – Not at all

1 – Once a week or less/a little

2 – 2 to 3 times a week/somewhat

3 – 4 to 5 times a week/very much

4 – 6 or more times a week/severe

15. Acting more irritable or aggressive with others

0 – Not at all

1 – Once a week or less/a little

2 – 2 to 3 times a week/somewhat

3 – 4 to 5 times a week/very much

4 – 6 or more times a week/severe

16. Taking more risks or doing things that might cause you or others harm (for example, driving recklessly, taking drugs, having unprotected sex)

0 – Not at all

1 – Once a week or less/a little

2 – 2 to 3 times a week/somewhat

3 – 4 to 5 times a week/very much

4 – 6 or more times a week/severe

17. Being overly alert or on-guard (for example, checking to see who is around you, being uncomfortable with your back to a door)

0 – Not at all

1 – Once a week or less/a little

2 – 2 to 3 times a week/somewhat

3 – 4 to 5 times a week/very much

4 – 6 or more times a week/severe

18. Being jumpy or more easily startled (for example when someone walks up behind you)

0 – Not at all

1 – Once a week or less/a little

2 – 2 to 3 times a week/somewhat

3 – 4 to 5 times a week/very much

4 – 6 or more times a week/severe

19. Having trouble concentrating

0 – Not at all

1 – Once a week or less/a little

2 – 2 to 3 times a week/somewhat

3 – 4 to 5 times a week/very much

4 – 6 or more times a week/severe

20. Having trouble falling or staying asleep

0 – Not at all

1 – Once a week or less/a little

2 – 2 to 3 times a week/somewhat

3 – 4 to 5 times a week/very much

4 – 6 or more times a week/severe

**DISTRESS AND INTERFERENCE**

21. How much have these difficulties been bothering you?

0 – Not at all

1 – Once a week or less/a little

2 – 2 to 3 times a week/somewhat

3 – 4 to 5 times a week/very much

4 – 6 or more times a week/severe

22. How much have these difficulties been interfering with your everyday life (for example relationships, work, or other important activities)?

0 – Not at all

1 – Once a week or less/a little

2 – 2 to 3 times a week/somewhat

3 – 4 to 5 times a week/very much

4 – 6 or more times a week/severe

**SYMPTOM ONSET AND DURATION**

23. How long after your childbirth experience did these difficulties begin? [choose one]

a. Less than 6 months

b. More than 6 months

24. How long have you had these childbirth experience-related difficulties? [choose one]

a. Less than 1 month

b. More than 1 month
