# Supplementary material for: Band of mothers: Childbirth as a female bonding experience
Source: PLoS One. 2020 Oct 21;15(10):e0240175. doi: 10.1371/journal.pone.0240175 (PMC7577500; doi:10.1371/journal.pone.0240175)
Supplement: S6 Appendix — (DOCX) [file pone.0240175.s006.docx]

**S6 Appendix. Adapted Version of the Posttraumatic Growth Inventory for the Postpartum Questionnaire.**

Please indicate for each of the statements below the degree to which change occurred in your life as a result of your experience of childbirth, using the following scale. (Scale of **0 – 5**, **0** being “I did not experience this change as a result of my experience” and **5** being “I experienced this change to a very great degree as a result of my experience”. Intermediate scores being **1** “a very small degree”, **2** “a small degree”, **3** “a moderate degree”, and **4** “a great degree”.)

1. My priorities about what is important in life.

**0** I did not experience this change as a result of my experience

**1** A very small degree

**2** A small degree

**3** A moderate degree

**4** A great degree

**5** I experienced this change to a very great degree as a result of my experience

2. An appreciation for the value of my own life.

**0** I did not experience this change as a result of my experience

**1** A very small degree

**2** A small degree

**3** A moderate degree

**4** A great degree

**5** I experienced this change to a very great degree as a result of my experience

3. I developed new interests.

**0** I did not experience this change as a result of my experience

**1** A very small degree

**2** A small degree

**3** A moderate degree

**4** A great degree

**5** I experienced this change to a very great degree as a result of my experience

4. A feeling of self-reliance.

**0** I did not experience this change as a result of my experience

**1** A very small degree

**2** A small degree

**3** A moderate degree

**4** A great degree

**5** I experienced this change to a very great degree as a result of my experience

5. A better understanding of spiritual matters.

**0** I did not experience this change as a result of my experience

**1** A very small degree

**2** A small degree

**3** A moderate degree

**4** A great degree

**5** I experienced this change to a very great degree as a result of my experience

6. Knowing that I can count on people in times of trouble.

**0** I did not experience this change as a result of my experience

**1** A very small degree

**2** A small degree

**3** A moderate degree

**4** A great degree

**5** I experienced this change to a very great degree as a result of my experience

7. I established a new path for my life.

**0** I did not experience this change as a result of my experience

**1** A very small degree

**2** A small degree

**3** A moderate degree

**4** A great degree

**5** I experienced this change to a very great degree as a result of my experience

8. A sense of closeness with others.

**0** I did not experience this change as a result of my experience

**1** A very small degree

**2** A small degree

**3** A moderate degree

**4** A great degree

**5** I experienced this change to a very great degree as a result of my experience

9. A willingness to express my emotions.

**0** I did not experience this change as a result of my experience

**1** A very small degree

**2** A small degree

**3** A moderate degree

**4** A great degree

**5** I experienced this change to a very great degree as a result of my experience

10. Knowing I can handle difficulties.

**0** I did not experience this change as a result of my experience

**1** A very small degree

**2** A small degree

**3** A moderate degree

**4** A great degree

**5** I experienced this change to a very great degree as a result of my experience

11. I’m able to do better things with my life.

**0** I did not experience this change as a result of my experience

**1** A very small degree

**2** A small degree

**3** A moderate degree

**4** A great degree

**5** I experienced this change to a very great degree as a result of my experience

12. Being able to accept the way things work out.

**0** I did not experience this change as a result of my experience

**1** A very small degree

**2** A small degree

**3** A moderate degree

**4** A great degree

**5** I experienced this change to a very great degree as a result of my experience

13. Appreciating each day.

**0** I did not experience this change as a result of my experience

**1** A very small degree

**2** A small degree

**3** A moderate degree

**4** A great degree

**5** I experienced this change to a very great degree as a result of my experience

14. New opportunities are available which wouldn’t have been otherwise.

**0** I did not experience this change as a result of my experience

**1** A very small degree

**2** A small degree

**3** A moderate degree

**4** A great degree

**5** I experienced this change to a very great degree as a result of my experience

15. Having compassion for others.

**0** I did not experience this change as a result of my experience

**1** A very small degree

**2** A small degree

**3** A moderate degree

**4** A great degree

**5** I experienced this change to a very great degree as a result of my experience

16. Putting effort into my relationships.

**0** I did not experience this change as a result of my experience

**1** A very small degree

**2** A small degree

**3** A moderate degree

**4** A great degree

**5** I experienced this change to a very great degree as a result of my experience

17. I’m more likely to try to change things which need changing.

**0** I did not experience this change as a result of my experience

**1** A very small degree

**2** A small degree

**3** A moderate degree

**4** A great degree

**5** I experienced this change to a very great degree as a result of my experience

18. I have a stronger religious faith.

**0** I did not experience this change as a result of my experience

**1** A very small degree

**2** A small degree

**3** A moderate degree

**4** A great degree

**5** I experienced this change to a very great degree as a result of my experience

19. I discovered that I’m stronger than I thought I was.

**0** I did not experience this change as a result of my experience

**1** A very small degree

**2** A small degree

**3** A moderate degree

**4** A great degree

**5** I experienced this change to a very great degree as a result of my experience

20. I learned a great deal about how wonderful people are.

**0** I did not experience this change as a result of my experience

**1** A very small degree

**2** A small degree

**3** A moderate degree

**4** A great degree

**5** I experienced this change to a very great degree as a result of my experience

21. I accept needing others.

**0** I did not experience this change as a result of my experience

**1** A very small degree

**2** A small degree

**3** A moderate degree

**4** A great degree

**5** I experienced this change to a very great degree as a result of my experience
