# Supplementary material for: Band of mothers: Childbirth as a female bonding experience
Source: PLoS One. 2020 Oct 21;15(10):e0240175. doi: 10.1371/journal.pone.0240175 (PMC7577500; doi:10.1371/journal.pone.0240175)
Supplement: S7 Appendix — (DOCX) [file pone.0240175.s007.docx]

**S7 Appendix. Adapted Version of the Event Related Rumination Inventory for the Postpartum Questionnaire.**

After an experience like the one you reported *(your experience of childbirth)*, people sometimes, but not always, find themselves having thoughts about their experience even though they don’t try to think about it. Indicate for the following items how often, if at all, you had the experiences described during the weeks immediately after your experience of childbirth. Scale of **0 – 3**, **0** = not at all to **3** = often.

I thought about the event when I did not mean to.

**0** Not at all

**1**

**2**

**3** Often

Thoughts about the event came to mind and I could not stop thinking about them.

**0** Not at all

**1**

**2**

**3** Often

Thoughts about the event distracted me or kept me from being able to concentrate.

**0** Not at all

**1**

**2**

**3** Often

I could not keep images or thoughts about the event from entering my mind.

**0** Not at all

**1**

**2**

**3** Often

Thoughts, memories, or images of the event came to mind even when I did not want them.

**0** Not at all

**1**

**2**

**3** Often

Thoughts about the event caused me to relive my experience.

**0** Not at all

**1**

**2**

**3** Often

Reminders of the event brought back thoughts about my experience.

**0** Not at all

**1**

**2**

**3** Often

I found myself automatically thinking about what had happened.

**0** Not at all

**1**

**2**

**3** Often

Other things kept leading me to think about my experience.

**0** Not at all

**1**

**2**

**3** Often

I tried not to think about the event, but could not keep the thoughts from my mind.

**0** Not at all

**1**

**2**

**3** Often

After an experience like the one you reported *(your experience of childbirth)*, people sometimes, but not always, deliberately and intentionally spend time thinking about their experience. Indicate for the following items how often, if at all, you deliberately spent time thinking about the issues indicated during the weeks immediately after your experience of childbirth. Scale of **0 – 3**, **0** = not at all to **3** = often.

I thought about whether I could find meaning from my experience.

**0** Not at all

**1**

**2**

**3** Often

I thought about whether changes in my life have come from dealing with my experience.

**0** Not at all

**1**

**2**

**3** Often

I forced myself to think about my feelings about my experience.

**0** Not at all

**1**

**2**

**3** Often

I thought about whether I have learned anything as a result of my experience.

**0** Not at all

**1**

**2**

**3** Often

I thought about whether the experience has changed my beliefs about the world.

**0** Not at all

**1**

**2**

**3** Often

I thought about what the experience might mean for my future.

**0** Not at all

**1**

**2**

**3** Often

I thought about whether my relationships with others have changed following my experience.

**0** Not at all

**1**

**2**

**3** Often

I forced myself to deal with my feelings about the event.

**0** Not at all

**1**

**2**

**3** Often

I deliberately thought about how the event had affected me.

**0** Not at all

**1**

**2**

**3** Often

I thought about the event and tried to understand what happened.

**0** Not at all

**1**

**2**

**3** Often
