# Supplementary material for: Band of mothers: Childbirth as a female bonding experience
Source: PLoS One. 2020 Oct 21;15(10):e0240175. doi: 10.1371/journal.pone.0240175 (PMC7577500; doi:10.1371/journal.pone.0240175)
Supplement: S8 Appendix — (DOCX) [file pone.0240175.s008.docx]

**S8 Appendix. Adapted Version of the Centrality of Event Scale for the Postpartum Questionnaire.**

Please think back upon your child’s birth and answer the following questions in an honest and sincere way, by choosing a number from **1 to 5**.

I feel that my child’s birth has become part of my identity.

**1** Totally disagree

**2**

**3**

**4**

**5** Totally agree

My child’s birth has become a reference point for the way I understand myself and the world.

**1** Totally disagree

**2**

**3**

**4**

**5** Totally agree

I feel that my child’s birth has become a central part of my life story.

**1** Totally disagree

**2**

**3**

**4**

**5** Totally agree

My child’s birth has colored the way I think and feel about other experiences.

**1** Totally disagree

**2**

**3**

**4**

**5** Totally agree

My child’s birth permanently changed my life.

**1** Totally disagree

**2**

**3**

**4**

**5** Totally agree

I often think about the effects my child’s birth will have on my future.

**1** Totally disagree

**2**

**3**

**4**

**5** Totally agree

My child’s birth was a turning point in my life.

**1** Totally disagree

**2**

**3**

**4**

**5** Totally agree
